# Regulatory Non-Coding RNAs Modulate Transcriptional Activation During B Cell Development

**DOI:** 10.3389/fgene.2021.678084

**Published:** 2021-10-14

**Authors:** Mary Attaway, Tzippora Chwat-Edelstein, Bao Q. Vuong

**Affiliations:** ^1^ Department of Biology, The City College of New York, New York, NY, United States; ^2^ Macaulay Honors College, New York, NY, United States; ^3^ The Graduate Center, The City University of New York, New York, NY, United States

**Keywords:** miRNA, lncRNA, lymphopoieis, hematopoiesis, B cell development and differentiation, transcription

## Abstract

B cells play a significant role in the adaptive immune response by secreting immunoglobulins that can recognize and neutralize foreign antigens. They develop from hematopoietic stem cells, which also give rise to other types of blood cells, such as monocytes, neutrophils, and T cells, wherein specific transcriptional programs define the commitment and subsequent development of these different cell lineages. A number of transcription factors, such as PU.1, E2A, Pax5, and FOXO1, drive B cell development. Mounting evidence demonstrates that non-coding RNAs, such as microRNAs (miRNAs) and long non-coding RNAs (lncRNAs), modulate the expression of these transcription factors directly by binding to the mRNA coding for the transcription factor or indirectly by modifying cellular pathways that promote expression of the transcription factor. Conversely, these transcription factors upregulate expression of some miRNAs and lncRNAs to determine cell fate decisions. These studies underscore the complex gene regulatory networks that control B cell development during hematopoiesis and identify new regulatory RNAs that require additional investigation. In this review, we highlight miRNAs and lncRNAs that modulate the expression and activity of transcriptional regulators of B lymphopoiesis and how they mediate this regulation.

## Introduction

**FIGURE 1:**
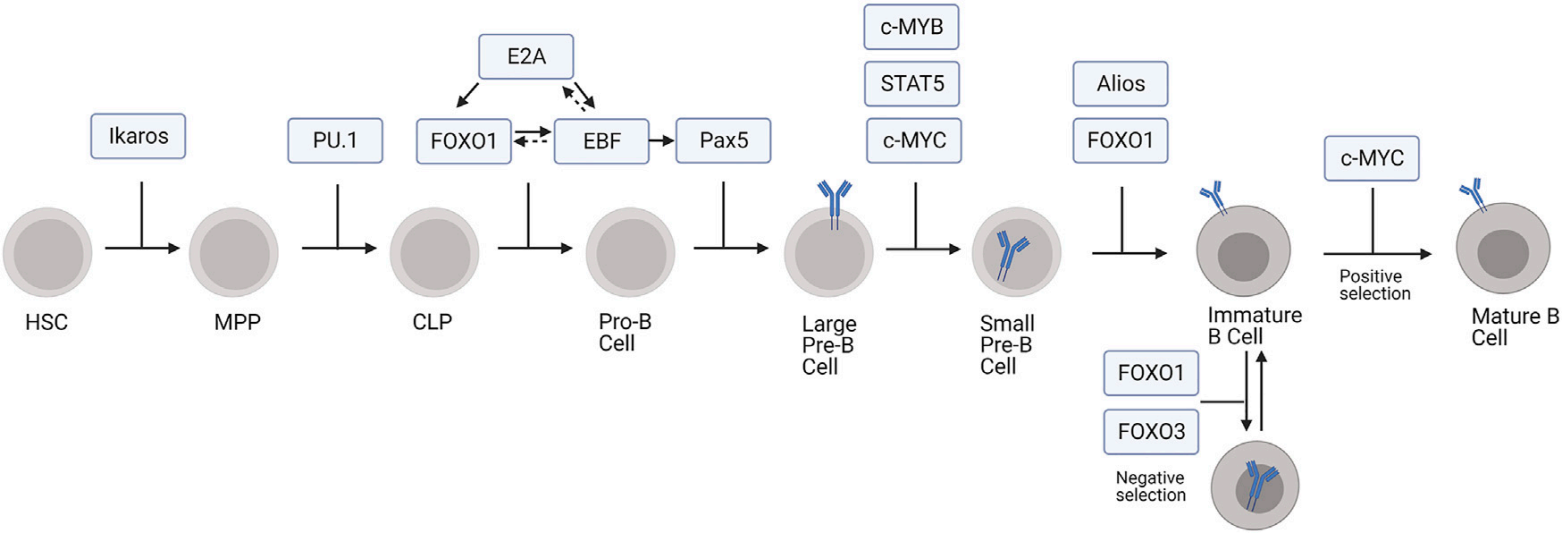
Transcription factors regulating development of B cells from HSCs. In this model, MPP includes both LMPP and ELP subpopulations.

Pluripotent, self-renewing hematopoietic stem cells (HSCs) differentiate into all blood cell types (Ng et al., 2009). Identified in the 1960s as a highly proliferative cell population in the bone marrow, HSCs give rise to myeloid, erythroid, megakaryocytic, and lymphoid cells (Till and McCulloch, 1961; Wu et al., 1967; Wu et al., 1968; Morrison et al., 1995). The lymphoid cells derived from HSCs, especially the T cells and B cells, are essential for the adaptive immune response. B cells implement the humoral immune response by producing immunoglobulins, which recognize and eliminate a wide variety of foreign antigens (Hoffman et al., 2016). To generate immunoglobulin producing B cells, HSCs develop into multipotent progenitors (MPPs) and common lymphoid progenitors (CLPs) before they commit to the B lineage (pro-B cells, pre-B cells, and immature B cells) (Figure 1) (Hardy and Hayakawa, 2001). During this latter developmental program, expression of specific transcription factors promote the sequential gene rearrangements at the immunoglobulin heavy chain (IgH) and light chain (IgL) gene loci (Fischer et al., 2020).

The first developmental transition from HSC to MPP is driven, at least in part, through the action of the transcription factor Ikaros (Schwickert et al., 2014). Ikaros suppresses stem cell and erythroid associated genes within MPPs, signaling the cells to begin differentiation and priming them to execute the lymphoid or myeloid developmental pathways (Ng et al., 2009). Ikaros also plays a role in guiding MPPs towards the lymphoid lineage through gene priming whereby Ikaros begins transcriptional activation of lymphoid specific genes, such as *Rag1*, before the cells formally commit to the lymphoid lineage (Pang et al., 2018). These MPPs, which are prepared to develop along the lymphoid lineage while still retaining some myeloid potential, are termed lymphoid-primed multipotent progenitors (LMPPs) (Nutt and Kee, 2007). The early lymphoid progenitors (ELPs) are another population within the LMPPs and can give rise to thymic progenitors for the T cell lineage or CLPs within the bone marrow for B cell development, natural killer cell development, and dendritic cell development (Nutt and Kee, 2007; De and Barnes, 2014). PU.1 promotes cytokine-dependent proliferation, survival, and thereby continued differentiation of MPPs into CLPs. Interestingly, the effect of PU.1 on MPP differentiation depends on its expression levels. High levels of PU.1 causes MPP cells to differentiate along the myeloid developmental pathway, whereas low levels cause them to develop into B cells (DeKoter and Singh, 2000). At lower concentrations, PU.1 promotes CLP development by activating lymphoid genes while also suppressing myeloid genes (Pang et al., 2018).

Pluripotent CLPs can also develop into T or B cells (Hardy and Hayakawa, 2001). B lineage commitment to the pro-B stage is marked by successive rounds of V(D)J recombination in the *IgH* gene locus, during which a variable (V), diversity (D), and joining (J) segment are joined together by the action of recombination activating genes 1 and 2 (*Rag1* and *Rag2*) (Hardy et al., 1991; Schatz et al., 1992). The pro-B cell developmental stage can be further divided into early pro-B cell and late pro-B cell stages wherein the D and J gene segments recombine followed by the V segment rearrangement with the previously established DJ segment, respectively (Hardy et al., 1991). The transition to the pro-B cell stage is largely mediated by the action of three transcription factors: E2A, Forkhead Box O1 (FOXO1), and Early B Cell factor (EBF) (Figure 1; Lin et al., 2010). First, E2A initiates the pro-B cell transcriptional program by activating expression of FOXO1 and EBF. E2A also activates *Rag1*, *Rag2*, and genes involved in pre-B cell receptor signaling, which positively selects for cells with productive rearrangement of *IgH* (Lin et al., 2010). Additionally, E2A represses genes responsible for the development of other lymphoid lineages and, through interactions with PU.1, myeloid lineages (Lin et al., 2010; Rogers et al., 2016).

FOXO1 in conjunction with E2A promotes the B lineage developmental program by upregulating EBF expression (Mansson et al., 2012). It also promotes IL-7Rα expression, which is necessary for pro-B cell survival (Dengler et al., 2008). EBF further supports the development of pro-B cell by promoting FOXO1 expression and activating genes for V(D)J recombination, including *Rag1* and *Rag2* (*Rag1/2*), and pre-B cell receptor (BCR) assembly, like VpreB (Hagman and Lukin, 2005; Mansson et al., 2012). Forkheadbox P1 (FOXP1) also regulates *Rag1/2* expression by binding to an enhancer region in the *Rag* locus (Hu et al., 2006). Additionally, EBF, likely in synergy with E2A and FOXO1, activates Pax5, which facilitates the transition from pro-B cell to pre-B cell (Revilla-I-Domingo et al., 2012). Subsequently, Pax5 allows for the pre-B cell transition by activating B cell specific genes, especially those involved in pre-BCR signaling, like *Cd19*, *mb-1*, *Blnk*, and *VpreB* (Hagman and Lukin, 2006). It also represses non-B lineage gene expression as well as genes associated with pluripotency (Pridans et al., 2008).

Pro-B cells develop into pre-B cells, which can be divided into two substages, large pre-B cells and small pre-B cells (25). Large pre-B cells express surface pre-BCR and undergo transient proliferation, which requires FOXO1 and FOXO3 phosphorylation and transcriptional inactivation to stop expression of genes required for V(D)J recombination, such as *Rag1/2*, and to allow for cell cycle reentry (Brunet et al., 1999; Amin and Schlissel, 2008; Li et al., 2019). Suppression of *Foxo1* and *Rag1/2* expression is also mediated by c-MYB, a transcriptional repressor that directly binds to regulatory sites of both *Foxo1* and *Rag* gene loci (Greig et al., 2008; Timblin et al., 2017). Cell cycle reentry and proliferation is further driven by c-MYC and IL7-receptor signaling via signal transducer and activator of transcription 5 (STAT5), which enhances transcription of cell-cycle effector cyclin D3 (CCND3) (Malin et al., 2010; Clark et al., 2014).

Pre-B cells exit the cell cycle and undergo IgL recombination during the small pre-B cell phase (Geier and Schlissel, 2006). Alios and B cell lymphoma 6 (BCL-6) prevent further proliferation and cell cycle progression by repressing the expression of *Myc, Ccnd3,* and *Ccnd2* (Mandal et al., 2009; Ma et al., 2010; Nahar et al., 2011). Transcriptional activation of IgL by E2A, PU.1, and interferon regulatory factor 4 (IRF4) promotes IgL V-J recombination, which is driven by the reactivation of the FOXO proteins and subsequent re-expression of *Rag1/2* (Herzog et al., 2008; Mandal et al., 2009; Batista et al., 2017). After small pre-B cells complete IgL recombination and begin to express surface BCR, they become immature B cells (Nemazee, 2017). Autoreactive cells expressing a BCR that recognizes self-antigens in the bone marrow promotes receptor editing or apoptosis, mechanisms of central tolerance (Nemazee, 2017). FOXO1 promotes receptor editing by inducing *Rag1/2* re-expression and consequently secondary V-J IgL recombination (Nemazee, 2006; Amin and Schlissel, 2008), whereas FOXO3 deletes autoreactive immature B cells through apoptotic pathways (Ottens et al., 2018). In the absence of BCR stimulation, transient tonic signaling *via* CD19 sequesters FOXO1 and FOXO3 to downregulate *Rag1/2* and directs the immature B cell to undergo positive selection and development to the transitional B cell stage (Monroe, 2006; Verkoczy et al., 2007).

The developmentally regulated activity of transcriptional activators and repressors during B lymphopoiesis generates a repertoire of B cells that recognizes and eliminates foreign antigens while ignoring self-antigens. Recent studies have identified non-coding microRNAs (miRNAs) and long non-coding RNAs (lncRNAs) in developing B lineage cells. In this review, we summarize the miRNAs and lncRNAs that control the expression of transcriptional regulators during B cell development.

## MicroRNAs in Early B Cell Development

MicroRNAs (miRNAs) are short non-coding RNAs between 19 and 23 nucleotides long that regulate protein expression (O'Brien et al., 2018). Most miRNAs post-transcriptionally repress target gene expression by binding to the 3′ untranslated region (UTR) of their target mRNA to inhibit mRNA translation and promote mRNA degradation (Pillai et al., 2007). Some miRNAs bind to regions outside of the 3′UTR of the target mRNA to repress translation, whereas others activate gene expression rather than repress (O'Brien et al., 2018). Processing of primary miRNAs transcripts by the ribonuclease III enzymes Drosha and Dicer with the RNA binding protein DiGeorge Syndrome Critical Region 8 (DGCR8) generates mature miRNAs, which may be derived from a cluster of miRNAs synthesized from a single transcript (Denli et al., 2004; O'Brien et al., 2018).

MiRNAs have been implicated as critical regulators of B cell development from the hematopoietic stem cell stage to mature B cells. Early studies showed that deletion of a floxed Dicer allele in mice with a cre recombinase under the control of the Mb1 promoter (Mb1-cre) blocks B cell development at the pro-B to pre-B cell transition (Koralov et al., 2008). A similar block in B cell development was observed following deletion of Drosha or DGCR8 with the Mb1-cre, suggesting that miRNAs rather than other non-coding RNAs regulate B cell developmental pathways (Coffre et al., 2016). Transcriptome analyses identified the prevalence of miRNAs throughout all stages of B cell development and showed distinct miRNA expression patterns at each stage of B cell development (Zhang et al., 2009; Kuchen et al., 2010; Spierings et al., 2011). Here, we focus on the miRNAs that modulate the activity of key transcription factors controlling B cell development.

**FIGURE 2:**
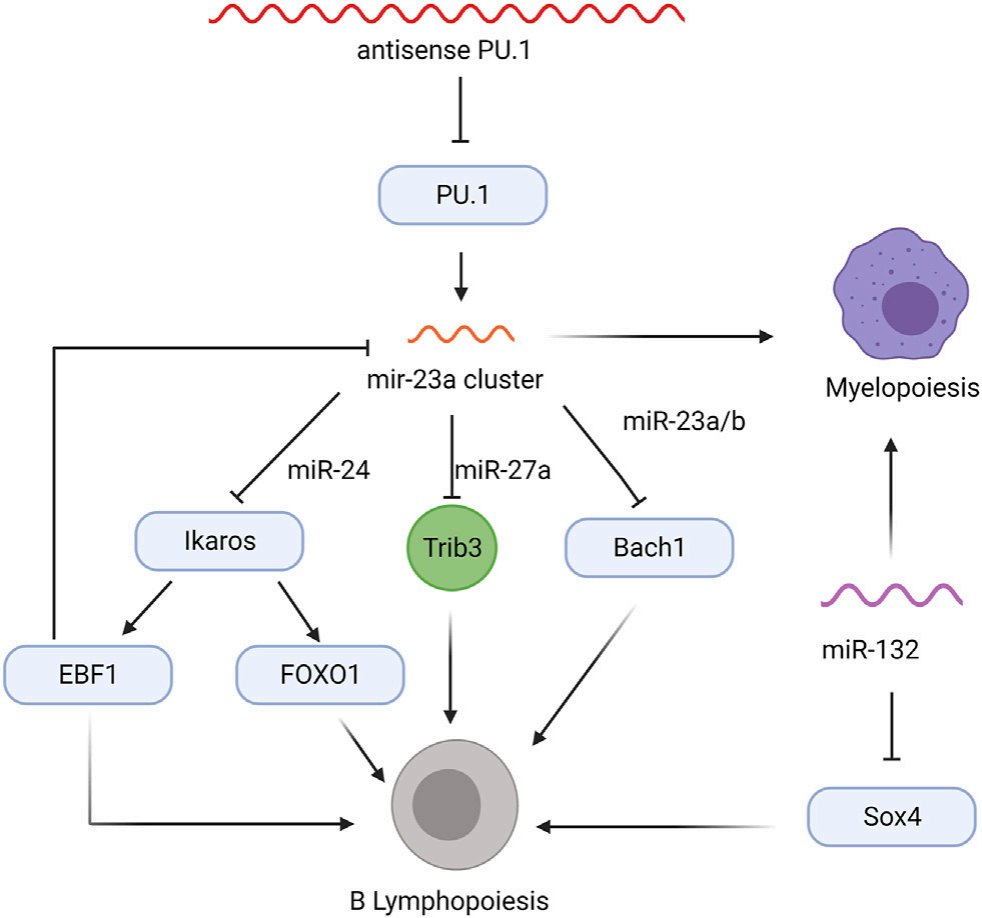
Non-coding RNA regulatory network in MPPs that influence myelopoiesis and B lymphopoiesis. Transcription factors are indicated in blue ovals, the Trib3 kinase is shown in green circle, and regulatory RNAs are colored wavy lines.

The miR-23a cluster, which includes miR-23a, miR-24-2, and miR-27a, prevents early B lineage commitment at the MPP stage (Kong et al., 2010; Kurkewich et al., 2016; Kurkewich et al., 2018; Kurkewich et al., 2017). Upregulated by PU.1, expression of the miR-23a cluster is predominantly observed in myeloid cells expressing high levels of PU.1, but is also observed at low levels in bone marrow cells negative for any lineage markers (Lin-) and CD19^+^ mature B cells expressing low levels of PU.1 (Kong et al., 2010). Overexpression of the miR-23a cluster, specifically miR-24, in cultured Lin^-^ bone marrow cells induces myelopoiesis over lymphopoiesis (Kong et al., 2010). Notably, similar results were observed after transferring miR-23a overexpressing HSCs into lethally irradiated mice (Kong et al., 2010). Consistent with these observations, *mirna23* ^−/−^ mice show increased CLP populations, Ly6D^+^ B-cell-biased lymphoid progenitors within the CLP population, and bone marrow B cells and decreased myeloid cells, highlighting the essential role of miR-23a in regulating myeloid and lymphoid lineage commitment (Kurkewich et al., 2016). Subsequent studies revealed that miRNAs of this cluster antagonize transcription factors required for B cell lymphopoiesis: miR-27a targets Ikaros, which promotes *Foxo1*, *Ebf1*, and *Pax5* expression during B lineage commitment, while miR-24 targets Trib3, a kinase promoting *Ebf1* and *Foxo1* expression by antagonizing Akt signaling, and miR23a/b targets *Bach1*, a transcriptional repressor of the myeloid program (Figure 2; Itoh-Nakadai et al., 2014; Kurkewich et al., 2016; Kurkewich et al., 2017). While higher levels of PU.1 upregulate the miR-23a cluster, transcription factors mediating B cell development negatively regulate miR-23a expression. E2A, EBF1, and Pax5 decrease *mirn23a* promoter activity in luciferase assays, but only EBF1 affected miR-23a, miR-24-2, and miR-27a levels in subsequent knockdown and overexpression assays (Kurkewich et al., 2017). These data suggest a role for the miR23a cluster in balancing the expression of various transcription factors that determine myeloid versus lymphoid cell fates and sustain MPP pluripotency by restricting the expression of B lineage commitment factors (Figure 2).

Early B lineage commitment is also regulated by the miR-212/132 cluster, which controls differentiation of CLPs into pro-B cells (Mehta et al., 2015). Lethally irradiated mice reconstituted with HSCs overexpressing miR-132, but not miR-212, show a severe decrease in all bone marrow B cells, accompanied by a slight but significant increase in myeloid cells (Mehta et al., 2015). Interestingly, *miR-212/132* ^*−/−*^ mice do not display any significant alteration in the frequencies of B cells or myeloid cells; however, depletion of mature B cells in *miR-212/132* ^*−/−*^ mice promotes B cell recovery and higher frequencies of pro-B, pre-B, and CD19^+^ B cells, suggesting the loss of miR-132 improves B cell recovery following inflammatory stress (Mehta et al., 2015). The miR-132 overexpressing bone marrow B cells show a reduction in both protein and mRNA levels of Sox4, a transcription factor required for pro-B cell development and survival (Sun et al., 2013; Mehta et al., 2015). The Sox4 3′UTR contains an miR-132 binding site and, consistent with this, miR-132 directly suppresses Sox4 transcription in reporter assays (Mehta et al., 2015). Co-overexpression of Sox4 and miR-132, which rescues the block in B cell development, confirmed the miR-132/Sox4 regulatory network in the earliest steps of B cell lineage commitment (Figure 2; Mehta et al., 2015); however, the factors modulating miR-132 expression remain unknown.

**FIGURE 3:**
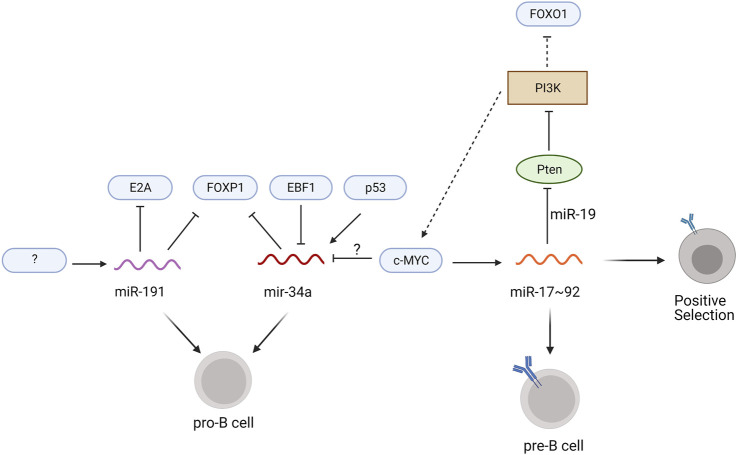
miRNAs regulating pro-B to pre-B development. Transcription factors are indicated in blue ovals, the phosphoinositide kinase PI3K is shown as a brown box, the PTEN phosphatase is shown as a green oval, and miRNAs are colored wavy lines. Solid lines specify the direct regulation of mRNAs by the miRNA and dotted lines designate modulation of signaling pathways that alter the expression of the transcription factors. Unidentified regulators are indicated with question marks.

Recently, Blume et al. identified miR-191 as a master regulator of transcription factors controlling the pro-B to pre-B cell transition. Transcriptome analysis revealed that miR-191 expression increases throughout B cell development with peak levels in the pro-B cell to immature B cell stages and lower levels in HSCs, MPPs, and mature B cells (Blume et al., 2019). *mir191* ^−/−^ mice showed a reduction in bone marrow B cells, specifically at the pro-B cell stage, and these pro-B cells have elevated FOXP1 and E2A mRNA levels (Blume et al., 2019), suggesting that mir191 downregulation of FOXP1 and E2A is necessary for the development of pro-B cells into pre-B cells (Figure 3). Conversely, chimeric mice overexpressing miR-191 in Lin^-^ bone marrow cells show a 40% reduction in bone marrow B cells and accumulate pro-B cells with a marked reduction of E2A and FOXP1 mRNA (Blume et al., 2019). The cells overexpressing miR-191 had decreased levels of *Rag1/2* transcripts and a deficiency in V to DJ recombination, both of which contribute to the developmental block at the pro-B cell phase (Blume et al., 2019). Interestingly, the defect in pro-B cell development and V-DJ recombination were rescued by overexpression of E2A, while only the pro-B cell developmental block was rescued by overexpression of FOXP1 (Blume et al., 2019), suggesting that FOXP1 functions beyond regulating *Rag1/2* expression to allow developing pro-B cells to bypass V-DJ recombination. Additional studies examining the transcriptional control of miR-191 expression and alternative mRNA targets of miR-191 will expand our understanding of the complex gene regulatory network in developing pro-B cells.

Another miRNA regulating pro-B cell development is miR-34a, which binds to the 3′UTR of FOXP1 mRNA to suppress translation (Rao et al., 2010). Constitutive expression of miR-34a in chimeric mice leads to a block in B cell development at the pro-B cell stage, while knockdown of miR-34a leads to increased numbers of mature bone marrow B cells (Rao et al., 2010). Transcriptional activation by p53 promotes miR-34a expression (Chang et al., 2007), whereas c-MYC knock down increases levels of miR-34a (Figure 3) (Craig et al., 2011; Zhai et al., 2020). Whether c-MYC directly represses miR-34a transcription remains to be determined. Luciferase reporter assays using the miR-34a promoter suggests that EBF1 may negatively regulate miR-34a transcription in diffuse large B cell lymphoma cells (Anastasiadou et al., 2019). Additional experiments in pro-B cells are required to further elucidate the role of EBF1 in miR-34a transcription during B cell development.

The miR-17–92 cluster, which includes miR-17, miR-18a, miR-19a/b, and miR-92, mediates the pro-B to pre-B cell transition as well as central tolerance (Psathas et al., 2013). Deletion of the entire miR-17–92 cluster using an Mb1-cre results in an accumulation of pro-B cells and increased apoptosis of pre-B cells (Lai et al., 2016). Ectopic expression of miR-17 rescues this pro-B cell developmental block but the mRNA target of miR-17 in pro-B cells remains elusive (Lai et al., 2016). Interestingly, c-MYC binding sites are located within the miR-17 gene locus and c-MYC induces expression of the miR-17–92 cluster in human fibroblasts as well as mature splenic B cells (O'Donnell et al., 2005; Benhamou et al., 2016).

Differentiation of pre-B cells into immature B cells is regulated in part by miR-150 (Xiao et al., 2007; Zhou et al., 2007). Expressed at low levels in pro-B and pre-B cells, miR-150 is upregulated in immature and mature B cells (Zhou et al., 2007). Noting the contrary expression pattern of c-MYB, which is expressed in B cell progenitors but not mature B cells,Xiao et al. (2007) showed that miR-150 directly targets the 3′UTR of c-MYB to suppress its expression. Consistent with this, lethally irradiated mice reconstituted with fetal liver HSCs overexpressing miR-150 display a developmental block at the pro-B cell stage and fail to produce normal numbers of pre-B, immature, and mature B cells (Zhou et al., 2007). Taken together, this indicates that premature expression of miR-150 leads to the suppression of c-MYB in pro-B cells, preventing their further differentiation. In normal B cells, however, miR-150 downregulates c-MYB at the immature B cell to permit development. The factors upregulating miR-150 remain uncharacterized.

At the immature B cell stage, miRNAs of the miR-17–92 cluster promote positive selection and B cell maturation (Lai et al., 2016). miR-19 suppresses phosphatase and tensin homolog (Pten), an upstream inhibitor of c-MYC and FOXO1 expression that antagonizes PI3K signaling (Lai et al., 2016). In immature B cells deficient for CD19, which are at heightened risk for activation-induced cell death, overexpression of the miR-17–92 cluster increases cell survival due to lower expression of Pten and higher expression of c-MYC, while ectopic expression of an miR-17–92 sponge increases activation-induced cell death (Benhamou et al., 2016). These discoveries led to a proposed positive feedback loop in which c-MYC upregulates its own expression through miR-17–92 induction (Figure 3; Benhamou et al., 2016). The enhanced PI3K signaling within this loop limits FOXO1 expression, which prevents receptor editing, and further promotes positive selection (Benhamou et al., 2018). Thus, miR-17–92 plays a crucial role in positive selection and the maturation of transitional B cells, in part through c-MYC-induced proliferation and reduced FOXO1-mediated receptor editing.

## Long Non-Coding RNAs in Hematopoiesis and Early B Cell Development

Transcription activates 70–90% of mammalian genomes; however, less than 3% of these transcripts are translated into proteins (Kashi et al., 2016). These untranslated transcripts include long non-coding RNAs (lncRNAs), which are 200 nucleotides or longer and contain no open reading frame (Kapranov et al., 2007). Like messenger RNAs (mRNAs), lncRNAs often contain poly-A tails and 5’ caps, which allows them to localize in both the nucleus and cytoplasm (Guttman et al., 2009; Alvarez-Dominguez and Lodish, 2017). Categorized by their position in relation to coding genes, lncRNAs include long intergenic RNA, long intronic RNA, antisense RNA, and pseudogene RNA (Satpathy and Chang, 2015). LncRNAs function *in cis* or *in trans* to alter gene expression by modulating chromatin structure, regulating transcription, and altering mRNA or protein stability (Rinn et al., 2007; Mao et al., 2011; Dimitrova et al., 2014; Hacisuleyman et al., 2014; Hu et al., 2014; Bao et al., 2015; Nobili et al., 2017; Winkle et al., 2017). Recent studies show that lncRNAs modulate the activity of essential transcription factors during hematopoiesis and B cell development.

Two characterized lncRNAs, lncHSC-1 and lncHSC-2, function early in hematopoiesis (Luo et al., 2015). Transcriptome analysis of HSCs, B cells, and granulocytes from bone marrow identified lncRNAs specifically enriched in HSCs (Luo et al., 2015). Subsequent fluorescent *in situ* hybridization (FISH) experiments revealed that lncHSC-1 and lncHSC-2 both predominantly localize in the nucleus, suggesting that they might regulate gene expression at the level of transcription rather than at a post-transcriptional or translational level (Luo et al., 2015). Knock-down of lncHSC-1 in Stem cell antigen-1 positive (Sca-1^+^) stem and progenitor cells increases myeloid lineage differentiation *in vivo* with a reduction in B and T lymphopoiesis, indicating that lncHSC-1 regulates MPP lineage commitment. Knock-down of lncHSC-2 in Sca-1^+^ stem cells increases peripheral T cell numbers while decreasing B cells (Luo et al., 2015), suggesting that lncHSC-2 determines ELP and/or CLP cell fate decisions. Interestingly, promoters for lncHSC-1 and lncHSC-2 contain binding sites for HSC-associated transcription factors, including PU.1, suggesting that their expression is developmentally regulated (Luo et al., 2015). Combinatorial analysis of lncHSC-2 ChIRP-seq (chromatin isolation by RNA purification sequencing) and E2A ChIP-seq (chromatin immunoprecipitation sequencing) data revealed common genomic binding sites for lncHSC-2 and E2A (Luo et al., 2015). Consistent with this, binding of E2A at lncHSC-2 genomic targets (*Nln*, *Slc35c2*, and *Itgb2*) was abrogated upon lncHSC-2 knock-down, suggesting that lncHSC-2 mediates E2A binding at these sites (Luo et al., 2015). These data emphasize the complex interplay between transcription factors and lncRNAs during hematopoiesis.

An antisense lncRNA modulates the expression of PU.1, an essential transcription factor for early B lineage development (Figure 1; DeKoter and Singh, 2000; Ebralidze et al., 2008). Knock-down of antisense PU.1 (asPU.1) increases PU.1 protein, suggesting that asPU.1 negatively regulates PU.1 expression (Figure 2; Ebralidze et al., 2008). Interestingly, RNA immunoprecipitation experiments revealed that knock down of asPU.1 increases PU.1 mRNA association with eF1A, an elongation factor that promotes translation, suggesting that asPU.1 modulates PU.1 expression through translational interference (Ebralidze et al., 2008). Thus, asPU.1 reduces levels of PU.1 to promote lymphopoiesis rather than myelopoiesis (DeKoter and Singh, 2000; Ebralidze et al., 2008; Pang et al., 2018).

Expression of lncRNAs occurs concomitantly with key transcription factors in developing B cells (Casero et al., 2015; Petri et al., 2015; Brazão et al., 2016). An exon array-based analysis of lncRNA expression in human bone marrow and tonsil samples identified distinct lncRNAs expression at various stages of human B cell development, which correlated with the expression of genes associated with each developmental stages, including *VpreB*, *Rag-1*, *Rag-2*, and *FLT3* (Petri et al., 2015). Interestingly, lncRNAs antisense to mRNAs encoding for transcription factors, such as *MYB, SMAD1,* and *LEF1*, which regulate B cell development during the pro-B cell to pre-B cell transition, were also identified (Reya et al., 2000; Thomas et al., 2005; Petri et al., 2015). Although the functional relevance of these lncRNAs remains unknown, other antisense lncRNAs, such as the HOX transcript antisense RNA (HOTAIR) and the aforementioned asPU.1, modulate the abundance or translation of their sense RNAs, suggesting a potential role for these lncRNAs in regulating *MYB*, *SMAD1*, and *LEF1* expression during B cell development (Rinn et al., 2007; Pelechano and Steinmetz, 2013; Petri et al., 2015). In a similar study, whole transcriptome sequencing identified and inferred functions for lncRNAs in developing human lymphocytes, implicating stage- and lineage-specific lncRNA expression in lymphoid commitment (Casero et al., 2015). Although both of the aforementioned studies catalog lncRNAs expressed uniquely in developing lymphocytes (LMPPs, CLPs, and B cell precursors), neither study ascribed a functional consequence or significance of lncRNA expression (Casero et al., 2015; Petri et al., 2015). A separate study identified lncRNAs expressed distinctly in developing mouse B cells (Brazão et al., 2016). Notably, many of the lncRNA gene loci contain Pax5 binding sites (Brazão et al., 2016). Analysis of RNA-seq data from Pax5-deficient cells further implicates a role for Pax5 in the expression of these lncRNAs and hence B cell development (Figure 1; Revilla-I-Domingo et al., 2012; Brazão et al., 2016). Interestingly, some of these mouse lncRNAs have human orthologs, suggesting evolutionarily conserved mechanisms regulating B cell development and/or function (Brazão et al., 2016). However, the functional significance and molecular targets of these lncRNAs in B cell development or function require further investigation.

## Concluding Remarks

The sequential action of specific transcription factors guides the development of B cells from HSCs. The function of transcription factors such as PU.1, E2A, and FOXO1 in B cell development have been well described (DeKoter and Singh, 2000; Dengler et al., 2008; Lin et al., 2010). However, the recent identification of non-coding RNAs, such as miRNAs and lncRNAs, underscore the complex genetic network promoting lineage commitment. Many of the aforementioned miRNAs, such as miR-23 and miR-132, modify transcription factor expression to promote B cell lineage commitment (Kong et al., 2010; Mehta et al., 2015). Similarly, lncHSC-1, lncHSC-2, and asPU.1 expression promotes B lineage development in the early stages of hematopoiesis (Ebralidze et al., 2008; Luo et al., 2015).

Despite the recent identification and characterization of non-coding RNAs in hematopoiesis and B lymphopoiesis, additional studies should elucidate their functional relevance during development. In particular, the molecular targets of the many evolutionarily conserved lncRNAs remain unknown (Zhang et al., 2009; Kuchen et al., 2010; Spierings et al., 2011; Casero et al., 2015; Petri et al., 2015; Brazão et al., 2016). The advent of deep sequencing technologies will aid our understanding of these molecular targets and pathways.

For example, ChIRP-seq can isolate areas of the genome bound by non-coding RNAs; crosslinking IP (CLIP) can identify protein-RNA interactions; and single cell sequencing can highlight developmental changes in gene expression (Chu et al., 2011; Thomson et al., 2011). Additionally, the ease of CRISPR gene editing could be used to analyze regions of non-coding RNAs that regulate gene expression or molecular pathways (Colognori et al., 2019; Tran et al., 2020). These experiments await further investigation but will certainly provide us with a unified understanding of hematopoiesis and B cell development.
